# Antioxidant Activities of Extract and Fractions from Receptaculum Nelumbinis and Related Flavonol Glycosides

**DOI:** 10.3390/ijms13067163

**Published:** 2012-06-11

**Authors:** Yan-Bin Wu, Li-Jun Zheng, Jian-Guo Wu, Ti-Qiang Chen, Jun Yi, Jin-Zhong Wu

**Affiliations:** 1 Academy of Integrative Medicine, Fujian University of Traditional Chinese Medicine, Fuzhou 350122, China; E-Mails: wxsq1@163.com (Y.-B.W.); zhenglijun1985@163.com (L.-J.Z.); wjg1419@126.com (J.-G.W.); 2 Fujian Academy of Agricultural Sciences, Fuzhou 350013, China; E-Mail: chen_tiqiang@189.cn; 3 Department of Chemistry and Life Science, Fujian Institute of Education, Fuzhou 350001, China; E-Mail: yijun1965@126.com

**Keywords:** *Nelumbo nucifera* Gaertn., receptacle, DPPH, ABTS

## Abstract

The antioxidant activities of ethanolic crude extract (ECE) and its four different solvent sub-fractions (namely, petroleum ether fraction (PEF), ethyl acetate fraction (EAF), *n*-butanol fraction (BF) and the aqueous fraction (AF) from the receptacles of *Nelumbo nucifera* Gaertn. (Receptaculum Nelumbinis) were investigated using two *in vitro* antioxidant assays. BF showed the highest total phenolic content (607.6 mg/g gallic acid equivalents), total flavonoid content (862.7 mg/g rutin equivalents) and total proanthocyanidin content (331.0 mg/g catechin equivalents), accompanied with the highest antioxidant activity compared to other fractions through 1,1-diphenyl-2-picrylhydrazyl (DPPH) and 2,2′-azino-bis-(3-ethylbenzthiazoline-6-sulphonic acid) (ABTS) radical scavenging assays. Five flavonol glycosides, namely hyperoside (**1**), isoquercitrin (**2**), quercetin-3-*O*-β-d-glucuronide (**3**), isorhamnetin-3-*O*-β-d-galactoside (**4**) and syringetin-3-*O*-β-d-glucoside (**5**) were isolated from the Receptaculum Nelumbinis. Compounds **2**–**5** were isolated for the first time from the Receptaculum Nelumbinis. The five isolated flavone glycosides, particularly compounds **1**–**3**, demonstrated significant DPPH and ABTS radical scavenging activity, with IC_50_ values of 8.9 ± 0.2, 5.2 ± 0.2, 7.5 ± 0.1 for DPPH and 114.2 ± 1.7, 112.8 ± 0.8, 172.5 ± 0.7 μg/mL for ABTS, respectively. These results suggest that Receptaculum Nelumbinis has strong antioxidant potential and may be potentially used as a safe and inexpensive bioactive source of natural antioxidants.

## 1. Introduction

*Nelumbo nucifera* Gaertn. (lotus) is an economically important aquatic plant grown in lakes, swamps, pools and rice fields. China is the leading lotus producer in the world; it has been cultivated for more than 2,000 years and is widely cultivated in almost all provinces including Hunan, Zhejiang, Fujian, Hubei, Anhui and Jiangxi [1]. Due its pleasant flavor and high nutritional value, *N. nucifera* is one of the most popular edible aquatic vegetables in China, especially its seed, rhizome and leaf, which have been widely used in Chinese cuisine and numerous food products like dessert, porridge, soup, drinks, and tea bags [2]. Moreover, almost all parts of *N. nucifera* are used for various medicinal purposes in oriental medicine.

Receptaculum Nelumbinis, called Lianfang in Chinese, is derived from the dried receptacle of *N. nucifera* and is commonly used in traditional Chinese medicine. It is used as an antihemorrhagic agent especially for excess menstrual bleeding and irregular genital bleeding, and also as a remedy for dehydration caused by diarrhea in summer and for prevention of miscarriage [3]. As reported in numerous previous studies, Receptaculum Nelumbinis does not only possess a substantial amount of phenolic compounds, but also exhibited a wide spectrum of biopharmacological effects, including antioxidation, improving learning and memory abilities, protective effects against experimental myocardial injury and ischemia, radioprotective activity, and anti-tumor effect [4–8].

All the above-mentioned biological activities focused on the polyphenol-enriched fraction of the Receptaculum Nelumbinis, suggesting that the phenolic constituents were the principle bioactive compounds existing in Receptaculum Nelumbinis. However, few phenolic compounds were reported from the medical material previously. According to our recent study, from among ten different parts of the lotus, the lotus receptacle showed not only the highest phenolic, flavonoid and proanthocyanidin contents, but also free-radical scavenging activities [4]. These results indicated that Receptaculum Nelumbinis contained an important quantity of antioxidant phytochemicals. Therefore, identification of the phenolic compounds must be carried out in order to continue with the mechanistic study of the antioxidant activity of Receptaculum Nelumbinis at the molecular and cellular levels.

This work was designed to evaluate the antioxidant properties of Receptaculum Nelumbinis extracts, and subsequently, to isolate potent antioxidant constituents. The structures of the isolated constituents were elucidated using spectral techniques (MS and NMR). Their in vitro antioxidant activities were assessed by the DPPH and ABTS scavenging methods as well. The data provide evidence for the utilization of Receptaculum Nelumbinis as a kind of herbal drug or food additive, as well as the possibility of using Receptaculum Nelumbinis as a safe and inexpensive bioactive source of natural antioxidants.

## 2. Results and Discussion

### 2.1. Total Flavonoids and Total Phenolic Content

The amounts of total phenolics, flavonoids, and proanthocyanidins in ethanolic crude extract (ECE) and its sub-fractions of Receptaculum Nelumbinis were determined, ranging from 32.1 to 607.6 mg/g of extract, from 42.8 to 862.7 mg/g of extract, and from 10.6 to 331.0 mg/g of extract, respectively. As shown in Table 1, the total phenolic content of CE and its fractions decreased in the following order: BF > ECE > EAF > AF > PEF, the total flavonoid content in the order of *n*-butanol fraction (BF) > ethyl acetate fraction (EAF) > ECE > aqueous fraction (AF) > petroleum ether fraction (PEF). A similar distribution was found in the total proanthocyanidin contents. From among four different polarity fractions, BF had the highest total phenolic, flavonoid, and proanthocyanidin contents and EAF came next. Furthermore, the total phenolic and total flavonoid contents were much higher than total proanthocyanidin of CE and its fractions.

### 2.2. Radical Scavenging Activities

**Figure 1:**
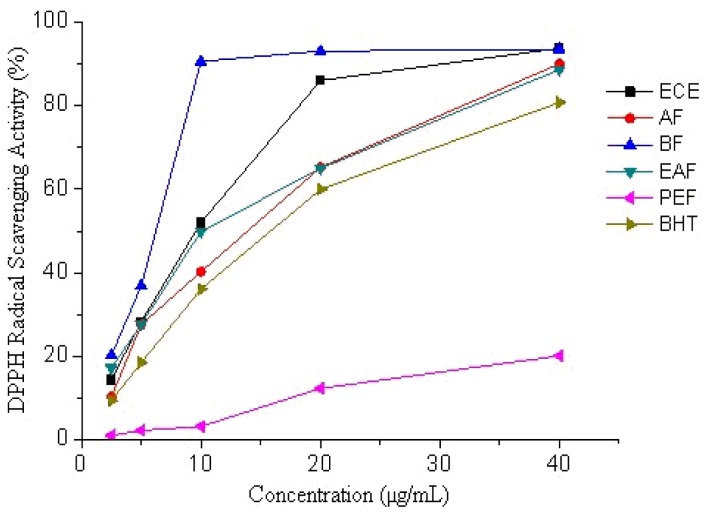
DPPH radical scavenging activity of ethanolic crude extract (ECE) and fractions of Receptaculum Nelumbinis (petroleum ether fraction (PEF), ethyl acetate fraction (EAF), *n*-butanol fraction (BF) and the aqueous fraction (AF)).

The radical scavenging activities of ECE and its fractions of Receptaculum Nelumbinis were evaluated using the methods of DPPH and ABTS radical scavenging activity assays, which have been widely used to test radical scavenging activity. As shown in Figure 1 and Table 2, the ECE and most of the sub-fractions showed obvious DPPH radical scavenging activity in a concentration-dependent manner. The scavenging ratios were improved with increasing sample concentration. Among the ECE of Receptaculum Nelumbinis and its fractions, BF (IC_50_ = 5.4 μg/mL) exhibited the most efficient radical scavenging activity, higher than those of the other fractions and ECE (Table 2). Except PEF, all fractions possessed significantly (*p* < 0.05) stronger radical scavenging ability than that of butylated hydroxyl toluene (BHT) (IC_50_ = 12.9 μg/mL).

**Figure 2:**
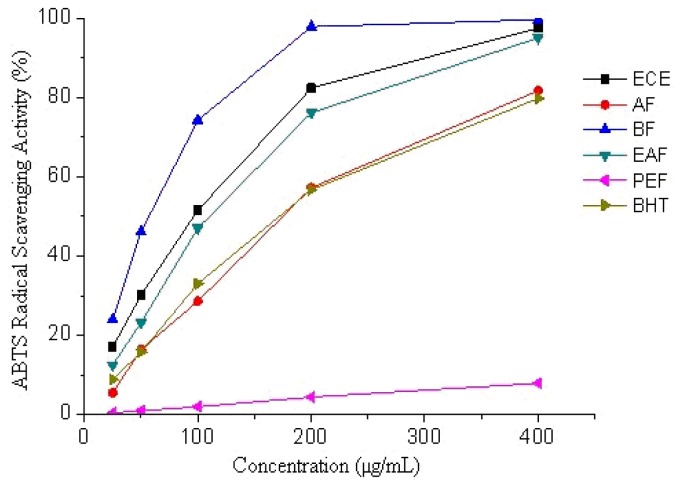
ABTS radical scavenging activity of ethanolic crude extract and fractions of Receptaculum Nelumbinis.

Similar scavenging activity patterns were seen in the ABTS assay. The ABTS radical scavenging activity of CE and its fractions increased with the increase of concentrations (Figure 2). It was observed that the BF (IC_50_ = 48.4 μg/mL) showed the highest radical scavenging activity of all the fractions (Table 2). ABTS radical scavenging activity of ECE and its fractions decreased in the following order: BF > EAF > ECE > BHT > AF > PEF (*p* < 0.05).

### 2.3. Structural Determination of the Isolated Compounds from *n*-Butanol Fraction (BF)

**Scheme 1:**
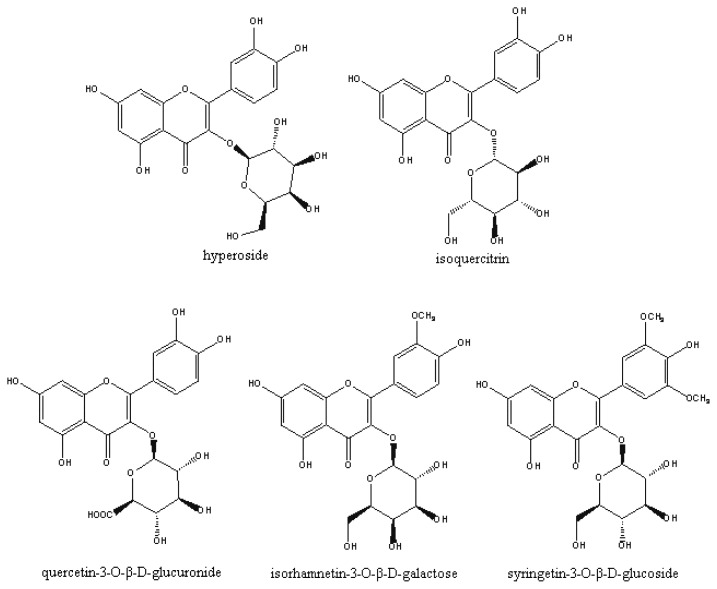
Structures of compounds isolated from Receptaculum Nelumbinis.

Based on the above results, activity-guided fractionation of the *n*-butanol fractions was carried out for the isolation of active constituents. Further fractionation and separation by silica gel and Sephadex LH-20 gel column chromatography methods yielded five flavonol glycosides. The structures of these compounds were identified as hyperoside (**1**), isoquercitrin (**2**), quercetin-3-*O*-β-d-glucuronide (**3**), isorhamnetin-3-*O*-β-d-galactoside (**4**) and syringetin-3-*O*-β-d-glucoside (**5**), respectively, by comparison of their spectroscopic data (^1^H, ^13^C NMR and MS) with those reported in the literature. Compounds **2**–**5** are isolated and identified for the first time from Receptaculum Nelumbinis. Their structures are shown in Scheme 1.

### 2.4. Antioxidant Activities of the Isolated Compounds from *n*-Butanol Fraction (BF)

Flavonoids are phenolic substances isolated from a wide range of vascular plants, with over 8000 individual compounds known, and have gained particular interest. Putative therapeutic effects of many traditional medicines may be ascribed to the presence of flavonoids, because of their broad pharmacological activity. The most important reported biological property of flavonoids is their antioxidant activity by scavenging radicals [9]. In this study the isolated compounds from *n*-butanol fraction of Receptaculum Nelumbinis were identified as five flavonol glycosides. The antioxidant activities of the five isolated flavonol glycosides were examined by using in the vitro antioxidant models described above, including DPPH and ABTS radical scavenging activities. As shown in Table 2, the scavenging effects of compounds **1**–**5** and BHT on DPPH and ABTS decreased in the following order: Compound **2** > Compound **1** > BHT > Compound **3** > Compound **4** > Compound **5** and Compound **2** > Compound **1** > BHT > Compound **3** > Compound **5** > Compound **4**, respectively. Among these tested compounds, isoquercitrin (Compound **2**) demonstrated highest DPPH and ABTS radical scavenging activities with IC_50_ values of 5.2 ± 0.2 and 112.8 ± 0.8 μg/mL, respectively. The DPPH radical scavenging activity of isoquercitrin was two times greater than that of the BHT, and ABTS radical scavenging activity of isoquercitrin was similar to that of the BHT. Compounds **1** and **3** exhibited effective DPPH and ABTS radical scavenging activity but inferior to isoquercitrin, while compounds **4** and **5** showed relatively weak activity.

It is interesting to investigate the structure-activity relationship for compounds **1**–**5**. Comparing their structures (Scheme 1), the main differences are the substituents at C-3′ and C-3. From comparison of the structures and activities of compounds **1**, **2** and **3**, it is inferred that the presence of a saccharide group at C-3 seems to have some, but little influence on antioxidant activity. By comparing compounds **1**, **2**, **3**, **4** and **5**, it is inferred that the presence of a –OH group at the C-3′ is the key factor that affects the activities. This is in agreement with previous studies: structure–activity relationship studies for the antioxidant efficacy of flavonoids have provided clear evidence that antioxidant activity depends on the catechol group in the B ring and the 2,3-double bond conjugated with the 4-oxo group [9].

Antioxidants are usually employed to prevent chronic and degenerative diseases by scavenging free radical intermediates or depressing the generation of free radicals [10,11]. It has been demonstrated that long-term treatment with Receptaculum Nelumbinis can prevent age-related increases in oxidation products and age-related deficits in cognitive functions that may be due to its free radical-scavenging and antioxidant activity, resulting from the presence of phenolic compounds in the extracts [12]. In addition, the antioxidant properties of Receptaculum Nelumbinis were believed to contribute to restore acetylcholine contents and acetylcholinesterase activities in the hippocampus and cerebral cortex of aged impaired animals [13]. The results of the present work also suggested that the activity of Receptaculum Nelumbinis could be explained, at least in part, by the presence of antioxidant flavonol glycosides. Further investigations, such as mechanistic studies of the antioxidant activity of flavonoids at both the molecular and cellular levels are underway.

## 3. Experimental Section

### 3.1. General

NMR spectra were obtained with a Bruker DRX-400 spectrometer at 400 MHz for ^1^H NMR and 100 MHz for ^13^C NMR. Peak positions are expressed in δ values with reference to TMS as internal standard, and coupling constants *J* in Hz. ESI-MS were recorded on a Varian MAT-212 mass spectrometer; column chromatography was performed on silica gel (200–300 mesh, Yantai, China) and Sephadex LH-20 (Pharmacia). The solvents used for isolation and purification were purchased from Sinopharm Chemical Reagent Co. Ltd. (Shanghai, China). Except butylated hydroxyl toluene (BHT) and aluminium trichloride, which were obtained from Sinopharm Chemical Reagent Co. (Shanghai, China), all other chemicals and reagents used in antioxidant activity assay like Folin-Ciocalteu reagent, 2,2′-diphenyl-1-picrylhydrazyl (DPPH), 2,2′-azino-bis(3-ethylbenzothiazoline-6-sulphonic acid) (ABTS), gallic acid and catechin were purchased from Sigma Chemical Co. (St. Louis, MO, USA). All other chemicals and solvents were of analytical grade.

### 3.2. Plant Material

Receptaculum Nelumbinis were collected from Fujian province of China and the voucher specimens were taxonomically identified based on morphological characteristics by J. Z. Wu and deposited in the Herbarium of the Academy of Integrative Medicine, Fujian University of Traditional Chinese Medicine in Fuzhou 350108, China.

### 3.3. Extraction and Isolation

The dried receptacles (21 kg) of *N. nucifera* were extracted with 75% ethanol two times under reflux, and the extracts were concentrated under reduced pressure. The residue obtained was suspended in water and successively partitioned with petroleum ether, ethyl acetate and *n*-BuOH, respectively. Each fraction was concentrated under reduced pressure to yield a petroleum ether fraction (PEF), an ethyl acetate fraction (EAF), a *n*-butanol fraction (BF) and a remainder (aqueous) fraction (AF). The *n*-BuOH extract (559 g) was subjected to silica gel column chromatography eluted with CH_2_Cl_2_-MeOH (50:1→30:1→20:1→10:1→5:1→3:1→1:1) and MeOH, and the resulting fractions were subjected to additional separation steps using a silica gel column and Sephadex LH-20 chromatography eluted with 85% methanol to yield compounds **1** (212 mg), **2** (180 mg), **3** (321 mg), **4** (26 mg) and Compound **5** (21 mg), respectively.

Hyperoside (**1**): Yellow powder; C_21_H_20_O_12_; ESI-MS: *m/z* 463 [M − H]^−; 1^H NMR (MeOD, 400 Hz): δ 5.15 (1H, d, *J* = 7.2 Hz, H-1″), 6.20 (1H, d, *J* = 2.0 Hz, H-6), 6.39 (1H, d, *J* = 2.0 Hz, H-8), 6.86 (1H, d, *J* = 8.4 Hz, H-5′), 7.58 (1H, dd, *J* = 2.0, 8.4 Hz, H-6′), 7.83 (1H, d, *J* = 2.0, H-2′). ^13^C NMR (MeOD, 100 MHz): δ 179.8 (C-4), 163.3 (C-5), 159.1 (C-7), 159.1 (C-9), 158.8 (C-2), 150.3 (C-4′), 146.1 (C-3′), 136.1 (C-3), 123.2 (C-1′), 123.2 (C-6′), 118.1 (C-5′), 116.4 (C-2′), 105.9 (C-10), 105.7 (C-1″), 100.3 (C-6), 95.1 (C-8), 77.5 (C-5″), 75.4 (C-3″), 73.5 (C-2″), 70.3 (C-4″), 62.5 (C-6″). These data are identical with those for Hyperin reported elsewhere [14].

Isoquercitrin (**2**): Yellow powder; C_21_H_20_O_12_; ESI-MS: *m/z* 463 [M − H]^−; 1^H NMR (MeOD, 400 Hz) δ 3.20–3.78 (5H, m, H-2″, 3″, 4″, 5″ and 6″), 5.24 (1H, d, *J* = 7.6 Hz, H-1″), 6. 23 (1H, d, *J* = 2.1 Hz, H-6), 6.42 (1H, d, *J* = 2.1 Hz, H-8), 6.88 (1H, d, *J* = 8.5 Hz, H-5′), 7.58 (1H, dd, *J* = 2.2, 8.5 Hz, H-6′), 7.72 (1H, d, *J* = 2.2 Hz, H-2′). ^13^C NMR (MeOD, 100 Hz) δ: 179.8 (C-4), 167.0 (C-7), 163.4 (C-5), 159.1 (C-2), 158.7 (C-9), 149.8 (C-4′), 145.9 (C-3′), 135.7 (C-3), 123.2 (C-1′), 123.1 (C-6′), 117. 6 (C-5′), 116.0 (C-2′), 105.8 (C-10), 104.4 (C-1″), 99.9 (C-6), 94.8 (C-8), 78.8 (C-5″), 78.2 (C-3″), 75.8 (C-2″), 71.3 (C-4″), 62.4 (C-6″). These data are identical with those for isoquercitrin reported elsewhere [15].

Quercetin-3-*O*-β-d-glucuronide (**3**): Yellow powder; C_21_H_27_O_13_; ESI-MS: *m/z* 477 [M − H]^−; 1^H NMR (MeOD, 400 Hz) δ: 8.08 ( 1H, br s, H-2′), 7.43 ( 1H, d, *J* =8.1 Hz, H-6′), 6.83 (1H, d, *J* = 8.1 Hz, H-5′), 6.37 (1H, br s, H-8), 6.19 (1H, br s, H-6), 5.33 (1H, d, *J* = 6.5 Hz, H-1″). ^13^C NMR (MeOD, 100 MHz) δ: 177.6 (C-4), 172.7 (C-6″), 164.7 (C-7), 161.0 (C-5), 157.1 (C-2), 156.5 (C-9), 148.5 (C-4′), 144. 9 (C-3′), 133. 8 (C-3), 121.1 (C-1′), 120.6(C-6′), 117.3 (C-2′), 115.4 (C-5′), 103.7 (C-10), 102.3 (C-1″), 99.0 (C-6), 93.7 (C-8), 76.5(C-3″), 74.7 (C-5″), 74.0 (C-2″), 71.8 (C-4″). These data are identical with those for quercetin-3-*O*-β-d-glucuronide reported elsewhere [16].

Isorhamnetin-3-*O*-β-d-galactoside (**4**): Yellow powder; C_21_H_17_O_13_; ESI-MS: *m/z* 477 [M − H]^−; 1^H NMR (DMSO-*d*_6_, 400 MHz): δ 8.04(1H, d, *J* = 2.0 Hz, H-2′), 7.51(1H, dd, *J* = 2.0, 9.0 Hz, H-6′), 6.90 (1H, d, *J* = 9.0 Hz, H-5′), 6.46 (1H, d, *J* = 2.0 Hz, H-8), 6.22 (1H, d, *J* = 2.0 Hz, H-6), 5.50 (1H, d, *J* = 7.5 Hz, H-1″). ^13^C NMR (DMSO-*d*_6_, 100 MHz): δ 177.6 (C-4), 164.4 (C-7), 161.4 (C-5), 156.5 (C-2), 156.4 (C-9), 149.6 (C-4′), 147.2 (C-3′), 133.2 (C-3), 122.0 (C-1′), 121.2 (C-6′), 115.3 (C-5′), 113.6 (C-2′), 104.1 (C-10), 101.8 (C-1″), 98.9 (C-6), 93.9 (C-8), 76.0 (C-5″), 73.3 (C-3″), 71.4 (C-2″), 68.1 (C-4″), 57.0 (3′-OCH_3_), 60.5 (C-6″). These data are identical with those for isorhamnetin-3-*O*-β-d-galactoside reported elsewhere [17].

Syringetin-3-*O*-β-d-glucoside (**5**): Yellow powder; C_23_H_24_O_13_; ESI-MS: *m/z* 507 [M − H]^−; 1^H NMR (MeOD, 400 Hz): δ 7.51 (2H, s, H-2′, 6′), 6.14 (1H, d, *J* = 2.0 Hz, H-8), 6.00 (1H, d, *J* = 2.0 Hz, H-7), 5.23 (1H, d, *J* = 7.0 Hz, H-1″), 3.91 (6H, s, 3′,5′-OCH_3_). ^13^C NMR (MeOD, 100 MHz): δ 178.4 (C-4),165.1 (C-7), 162.7 (C-5), 159.5 (C-9), 157.4 (C-2), 149.3 (C-3′,5′), 141.0 (C-4′), 135.4 (C-3), 121.4 (C-1′), 108.3 (C-2′,6′), 104.7 (C-6), 103.6 (C-1″), 103.1(C-10), 97.5 (C-8), 78.8 (C-5″), 78.5 (C-3″), 76.3 (C-2″), 71.8 C-4″), 62.8 (C-6″), 57.4 (3′,5′-OCH_3_). These data are identical with those for syringetin-3-*O*-β-d-glucoside reported elsewhere [18].

### 3.4. Determination of Total Phenols, Total Flavonoids and Total Proanthocyanidin

The total phenolic content of the crude extract and its fractions were determined using Folin-Ciocaleteu assay according to the previously described method [19]. Gallic acid (GA) was used as standard and the results were calculated as gallic acid equivalents (mg/g) of lotus receptacle extract through the calibration curve of gallic acid. The total flavonoid content was determined using a colorimetric assay according to the previously described method with some modifications [20]. The results were calculated and expressed as rutin equivalents (mg) per gram of lotus receptacle extract using the calibration curve of rutin. The total proanthocyanidin content was determined according to the modified previously described procedure [21], using (+)-catechin as a standard and the results were expressed as (+)-catechin equivalents in microgram per gram of lotus receptacles extract.

### 3.5. Evaluation of Antioxidant Activity

#### 3.5.1. DPPH Radical-Scavenging Activity

The DPPH radical scavenging activities of the crude extract of Receptaculum Nelumbinis and its fractions were determined according to the previously described method [4]. The DPPH radical scavenging activity was calculated by the following formula: Scavenging ability (%) = (1 − A_sample_/A_blank_) × 100%, where A_blank_ is the absorbance of the blank reaction (using 2 mL of methanol instead of the tested sample) and A_sample_ is the absorbance of the tested sample. For each sample, its concentration with 50% scavenging ability on the DPPH radicals (IC_50_) was determined by interpolation of linear regression analysis. BHT was used as positive control.

#### 3.5.2. ABTS Radical-Scavenging Activity

The ability of the crude extract and fractions of Receptaculum Nelumbinis to scavenge ABTS radicals were determined according to ABTS decolorization assay as previously described [4]. The ability of individual Receptaculum Nelumbinis extracts to scavenge ABTS radicals (%) and the IC_50_ were calculated as described in the DPPH radical scavenging assay. BHT was used as positive control.

### 3.6. Statistical Analysis

All the experiments were conducted in triplicates, and the data were presented as mean ± SD. SPSS (version 16.0; SPSS Inc.: Chicago, IL, USA, 2007) and Origin (version 8.0; Microcal Software Inc.: Northampton, MA, USA, 2007) were used to process the results, which were analyzed by one-way ANOVA and Tukey-HSD at *p* < 0.05 to detect significant differences among groups.

## 4. Conclusions

This study was designed to evaluate the total phenolic, flavonoid and proanthocyanidin contents and antioxidant activity of ECE and its sub-fractions of Receptaculum Nelumbinis. The BF sub-fraction showed the highest total phenolic and flavonoids contents, exhibited the highest free-radical scavenging activities. Five flavonol glycosides were isolated and their antioxidant activities were evaluated using DPPH and ABTS models. Hyperoside (**1**), isoquercitrin (**2**) and quercetin-3-*O*-β-d-glucuronide (**3**) showed effective activity and the structure-activity relation investigation indicated that the catechol group in the B ring affected the antioxidant activity. Being the by-products of lotus food production, the utilization of Receptaculum Nelumbinis is still low: it is usually discarded as waste. The obvious antioxidant activity in Receptaculum Nelumbinis confirmed its important role in the bioactivity of whole lotus and suggested that the Receptaculum Nelumbinis removal may induce a more significant nutrient loss. Until now, the Receptaculum Nelumbinis has not received much attention with respect to recycling it for the antioxidants instead of disposing it due to the lack of commercial application of these wastes. Our findings not only provide an added value to this regional bio-resource, but could also facilitate its development as a safe and inexpensive bioactive source of natural antioxidants, especially for the fast growing functional food industry nowadays.

## Figures and Tables

**Table 1:** Contents of total phenolics, flavonoids, and proanthocyanidins of ethanolic crude extract and fractions of Receptaculum Nelumbinis. ECE, ethanolic crude extract; PEF, petroleum ether fraction; EAF, ethyl acetate fraction; BF, *n*-butanol fraction; AF, aqueous fraction.

Sample	Total Phenolic Content (mg/g Extract)	Total Flavonoid Content (mg/g Extract)	Total Proanthocyanidin Content (mg/g Extract)
ECE	480.5 ± 12.6	507.4 ± 16.9	297.7 ± 6.3
PEF	32.1 ± 0.1	42.8 ± 0.2	10.6 ± 1.0
EAF	391.2 ± 2.6	637.5 ± 26.6	129.9 ± 6.5
BF	607.6 ± 4.0	862.7 ± 29.1	331.0 ± 6.4
AF	376.6 ± 8.7	241.5 ± 8.5	92.3 ± 4.4

**Table 2:** IC_50_ values (μg/mL) obtained in the antioxidant assays of DPPH scavenging and ABTS scavenging of crude extract and fractions of Receptaculum Nelumbinis.

Sample	DPPH Radical Scavenging Activity	ABTS Radical Scavenging Activity
ECE	7.6 ± 0.1	73.6 ± 3.7
PEF	103.4 ± 4.4	>500
EAF	9.4 ± 0.6	90.8 ± 4.2
BF	5.4 ± 0.3	48.4 ± 1.8
AF	12.0 ± 0.7	160.2 ± 2.4
Compound 1	8.9 ± 0.2	114.2 ± 1.7
Compound 2	5.2 ± 0.2	112.8 ± 0.8
Compound 3	7.5 ± 0.1	172.5 ± 0.7
Compound 4	193.1 ± 0.3	436.6 ± 4.2
Compound 5	286.6 ± 3.5	283.0 ± 1.5
BHT	12.9 ± 0.3	157.9 ± 1.4
